# The EGFR regulates bacterial clearance in cystic fibrosis airway neutrophils

**DOI:** 10.1172/JCI198292

**Published:** 2026-04-14

**Authors:** Lawrence W. Rasmussen, Deepali Luthra, Diego Moncada-Giraldo, Crystal Lewis, Yixel M. Soto-Vazquez, Zhuo Li, Buqu Hu, Brian S. Dobosh, Delores A. Stacks, Jonathan L. Koff, Amit Gaggar, Rabindra Tirouvanziam, Camilla Margaroli

**Affiliations:** 1 Department of Medicine, University of Alabama at Birmingham, Birmingham, Alabama, USA.; 2 Department of Pediatrics, Emory University, Atlanta, Georgia, USA.; 3 Department of Pathology, University of Alabama at Birmingham, Birmingham, Alabama, USA.; 4 Department of Medicine, Yale University, New Haven, Connecticut, USA.; 5 Birmingham VA Medical Center, Birmingham, Alabama, USA.

**Keywords:** Immunology, Pulmonology, Bacterial infections, Growth factors, Neutrophils

## Abstract

The epidermal growth factor receptor influences how airway neutrophils clear bacteria in cystic fibrosis, revealing a novel mechanism in neutrophil biology.

**To the Editor:** Cystic fibrosis (CF) lung disease is characterized by chronic inflammation and persistent bacterial infections despite the recent introduction of CFTR modulator therapies (elexacaftor, tezacaftor, and ivacaftor; ETI). A limiting factor hindering effective targeting of neutrophilic inflammation is the lack of knowledge of underlying mechanisms. Previously, we showed that neutrophils recruited to CF airways acquire the GRIM phenotype, featuring Granule Release, Immune modulation, and Metabolic activation, and reduced bacterial killing capacity (1) controlled by de novo transcription (2).

The epidermal growth factor receptor (EGFR) is implicated in several pathways that overlap with the GRIM phenotype. While the role of EGFR in the airway epithelium is well documented, EGFR expression in neutrophils is less established. Here, we investigated EGFR expression and its role in CF airway neutrophils.

Airway neutrophils from people with CF (pwCF) (Supplemental Table 1; Supplemental Figure 1, A and B; and Figure 1A; supplemental material available online with this article; https://doi.org/10.1172/JCI198292DS1) showed a median 2.5-fold increase in surface EGFR expression (Figure 1B), as well as phosphorylation of EGFR, suggesting activation and signaling (Figure 1C). Furthermore, we observed that treatment with ETI is likely not modulating EGFR expression in CF airway neutrophils (Supplemental Figure 1, D and E).

Next, we generated CF airway neutrophils in vitro (Supplemental Figure 1C) by transmigrating isolated healthy blood neutrophils through an airway epithelium biomimetic model, which allows for full recapitulation of airway neutrophil GRIM phenotype from pwCF independently of neutrophil origin (2). Blood neutrophils were transmigrated into leukotriene B_4_ (LTB_4_; control chemoattractant) or CF airway sputum supernatant (CFASN; devoid of cells and bacteria) (2, 3). RNA profiling showed that CF airway neutrophils had increased expression of *EGFR* and genes related to its intracellular signaling intermediates (Figure 1D) compared with blood and of its ligands compared with blood or LTB_4_ controls (Supplemental Figure 2).

Temporal analysis showed upregulation of EGFR expression in healthy blood neutrophils transmigrated into CFASN (Figure 1, E and F), which was confirmed by ELISA (Figure 1G), while EGFR phosphorylation in the same neutrophils revealed delayed activation (Figure 1H).

The EGF was the most abundant EGFR ligand in CF sputum (Supplemental Figure 3A), and it was selected to evaluate EGFR signaling. EGF stimulation of CF airway neutrophils increased intracellular calcium (Figure 1I), and EGFR activation in CFASN-transmigrated healthy neutrophils increased killing of *P*. *aeruginosa* in 4 out of 6 samples (Figure 1J, and Supplemental Figure 3, B and C), while EGFR inhibition abrogated it (Figure 1J). This heterogenous response should be further investigated in a larger study. This was confirmed with CF blood neutrophils transmigrated into CFASN (Supplemental Figure 3D). We confirmed the role of EGFR signaling in neutrophil bacterial clearance within CFASN in transmigrated healthy and CF neutrophils (Supplemental Figure 3E). While blockade of EGFR reduced their ability to clear *P*. *aeruginosa*, the baseline ability to clear planktonic bacteria was found to be incongruent with chronic infection observed in patients with CF. This discrepancy could be attributable to the use of reference bacterial strains, and future testing with clinical isolates or biofilm models will be required. Next, we investigated the role of EGFR-induced NOS in our experimental model. Surprisingly, treatment with EGF overcame pharmacological blockade of neuronal, inducible, and endothelial NOS to increase *P*. *aeruginosa* clearance (Supplemental Figure 3F). Given these findings and the fact that *S*. *aureus*, another common CF sputum pathogen, deploys mechanisms to evade host NO production, we evaluated if EGF stimulation of CF airway neutrophils would also increase bactericidal capacity against *S*. *aureus*. As observed with *P*. *aeruginosa*, EGF stimulation of CF airway neutrophils increased their capacity to clear *S*. *aureus* (Figure 1K). Last, stimulation of CFASN-transmigrated neutrophils with EGF in the presence of bacteria increased tyrosine nitrosylation (Figure 1L), indicative of an increase in myeloperoxidase activity downstream of EGFR.

Overall, the data presented here provide evidence for a role of EGFR signaling in CF airway neutrophils.

Indeed, it has been shown that EGFR signaling in macrophages is crucial for their activation and optimal downstream T cell responses (3). Beyond macrophages, airway epithelial cells may also respond to neutrophil-generated EGFR ligands. As EGFR expression is elevated in CF airway epithelium (4), neutrophil-derived ligands may contribute to chronic activation of epithelial inflammation promoting progressive lung disease (4). Taken together, EGFR-driven immune responses in both macrophages and airway epithelial cells suggest that neutrophil-mediated EGFR signaling may serve as a nexus that regulates immune and inflammatory processes in CF airways.

## Author contributions

CM, RT, and AG conceived the study. LWR, DL, CL, YMSV, ZL, BSD, and DAS performed the experiments for the study. JLK and BH provided expertise on EGFR biology and data analysis. DMG performed transcriptional analyses. CM and LWR wrote the manuscript. All authors revised the manuscript. Co–first authorship was determined by the extent of the contribution of each author to the manuscript. LWR performed the experiments and wrote the manuscript, and DL performed the experiments for NOS inhibition and for the revised manuscript.

## Conflict of interest

The authors have declared that no conflict of interest exists.

## Funding support

This work is the result of NIH funding, in whole or in part, and is subject to the NIH Public Access Policy. Through acceptance of this federal funding, the NIH has been given a right to make the work publicly available in PubMed Central.

- Cystic Fibrosis Foundation Postdoc-to-Faculty Award
- MARGAR21F5 (CM).
- NIH R01 HL159058 (RT).

## Supplementary Material

Supplemental data

Supporting data values

## Figures and Tables

**Figure 1:**
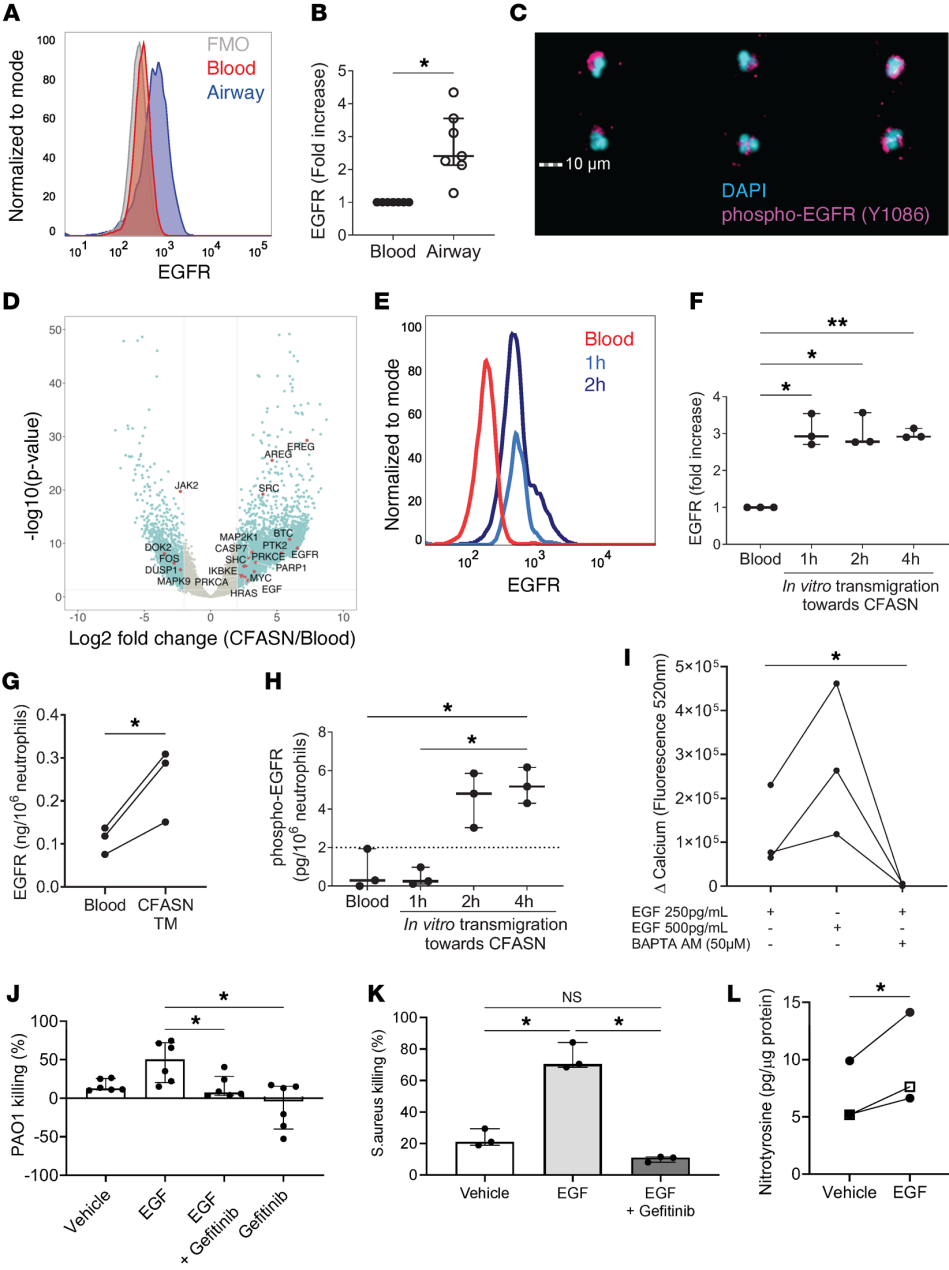
EGFR increases CF airway neutrophils’ bactericidal capacity. (**A**) EGFR expression in blood (shown in red) and airway (shown in blue) neutrophils from stable CF patients. FMO, fluorescence minus one. (**B**) EGFR expression on paired airway and blood neutrophils from CF patients (*N* = 7, data normalized to blood). (**C**) Representative Imagestream analysis of phosphorylated EGFR at tyrosine 1086 (Y1086, shown in magenta) in primary CF airway neutrophils (DAPI, shown in blue). (**D**) Healthy blood neutrophils transmigrated into CFASN for 10 hours upregulate EGFR and its ligands compared with matched blood neutrophils (*N* = 5). (**E**) EGFR expression in healthy blood neutrophils before transmigration (blood) and 1 hour and 2 hours after transmigration. (**F**) Fold increase in expression of surface EGFR between paired healthy blood and CFASN-transmigrated neutrophils (*N* = 3, data normalized to blood). (**G**) EGFR expression by ELISA on matched healthy blood and CFASN-transmigrated neutrophils. (**H**) EGFR phosphorylation in healthy CFASN-transmigrated neutrophils by ELISA. (**I**) Intracellular calcium levels in transmigrated CF neutrophils treated with EGF. (**J** and **K**) Clearance of *P*. *aeruginosa* and *S*. *aureus* by healthy CFASN-transmigrated neutrophils treated with EGF and/or gefitinib for 2 hours. (**L**) Quantification of nitrotyrosine in neutrophil lysates upon EGF stimulation and incubation with PAO1 (dots = CF neutrophils transmigrated into CFASN, open square = healthy control neutrophils transmigrated into CFASN). Statistical analysis was performed using Kruskal-Wallis with Dunn’s multiple-comparison test (**F** and **H**–**K**) or by Wilcoxon’s signed-rank test (paired; **B**, **G**, and **L**). *P < 0.05, **P < 0.01.

## Version 1 04/14/2026

In-Press Preview

## Version 2 06/15/2026

Electronic publication
