# Supplementary material for: The EGFR regulates bacterial clearance in cystic fibrosis airway neutrophils
Source: J Clin Invest. 2026 Apr 14;136(12):e198292. doi: 10.1172/JCI198292 (PMC13262706; doi:10.1172/JCI198292)
Supplement: Supplemental data [file jci-136-198292-s249.pdf]

The epidermal growth factor receptor regulates bacterial clearance in cystic fibrosis airway neutrophils

Lawrence W. Rasmussen[#1], Deepali Luthra[#2], Diego Moncada Giraldo[2], Crystal Lewis[3], Yixel M. Soto-Vazquez[1], Zhuo Li[2], Buqu Hu[4], Brian S. Dobosh[2], Delores A. Stacks[3], Jonathan L. Koff[4], Amit Gaggar[1,5], Rabindra Tirouvanziam[2], Camilla Margaroli[3*]

Supplemental materials
Methods
Tables: S1
Figures: S1-S3

Author contributions: CM, RT, and AG conceived the study; LWR, DL, CL, YMSV, ZL, BSD, DAS performed the experiments for the study; JLK and BH provided expertise on EGFR biology and data analysis; DMG performed transcriptional analyses; CM and LWR wrote the manuscript, all authors revised the manuscript. Co-first authorship was determined by the extent of the contribution of each author to the manuscript, LWR performed the experiments and wrote the manuscript, DL performed the experiments for NOS inhibition and for the revised manuscript.

Funding support: This work is the result of NIH funding, in whole or in part, and is subject to the NIH Public Access Policy. Through acceptance of this federal funding, the NIH has been given a right to make the work publicly available in PubMed Central. This work was funded by the Cystic Fibrosis Foundation Postdoc-to-Faculty Award MARGAR21F5 (C.M.), and R01 HL159058 (R.T.).

Methods

Sex as a biological variable

Given the small sample size for this study, sex was not considered as a biological variable, however both men and women were included in the study.

Patient samples

Blood was collected by venipuncture in K_2-EDTA tubes and used for profiling with flow cytometry or for in vitro transmigration experiments. Expectorated sputum was processed by mechanical dissociation as previously described (1) to obtain airway cells for flow cytometry phenotyping and CF airway supernatant (CFASN) for *in vitro* transmigration. Induced sputum was collected after instillation of hypertonic saline under supervision of a certified respiratory therapist and similarly processed to obtain airway cells for flow cytometry.

Flow cytometry

Single cell suspension in PBD-EDTA (2.5mM) were pre-incubated with FcX blocking agent and Live/Dead Zombie Aqua or nIR dyes (Biolegend) for 10 minutes on ice and in the dark. Staining for surface protein expression was then performed for 30 minutes on ice in the dark using antibodies against the following epitopes: CD66b (Biolegend, clone G10F5), CD63 (Biolegend, clone H5C6), CD16 (Biolegend, clone 3G8) epidermal growth factor receptor (EGFR) (Biolegend, clone AY13). After staining, cells were washed twice with 2mL of PBS-EDTA, resuspended and incubated overnight at 4°C in Lyse/Fix Phosflow (BD Biosciences). The fixative was removed by centrifugation at 800g for 10 minutes at 4°C and cells were resuspended in PBS-EDTA. Samples were acquired on the BD FACS Symphony or on a BD FACS LSRII with bead calibration to ensure constant output from the instruments across acquisitions. Data were analyzed in FlowJo v10.10.0 (BD Biosciences).

Image cytometry

Cells from expectorated sputum were fixed in Lyse/Fix Phosflow and stored at -80°C until use. Thawed cells were washed once with 2mL of PBS-EDTA, then permeabilized with Perm Buffer I (BD Biosciences) and stained with DAPI (nuclear stain) and an antibody against phospho-EGFR Y1086 (Cell Signaling Technologies, clone D7A5, cat#3777, conjugated to PE using the lightning-link kit from Novus Biologicals). Acquisition was performed on the Amnis Imagestream X Mark II (Cytek), with 40x magnification and low flow rate/high sensitivity on the INSPIRE software. Brightfield was set in channel 01, and scattering was set in channel 06. Data were analyzed using the IDEAS software v6.1 (Cytek).

ELISA

EGFR ligands were quantified in CFASN (from expectorated and induced sputum) by ELISA following manufacturer's protocols (R&D Systems). Transmigrated neutrophils were washed twice in cold PBS and lysed in RIPA buffer supplemented with 1X Protease HALT cocktail (Thermofisher) for 15 minutes on ice. Debris were removed by centrifugation at 14,000g for 15 minutes, and EGFR was quantified in the supernatant by ELISA following manufacturer's protocol (Invitrogen). Phospho-EGFR was quantified in transmigrated neutrophils by phospho-ELISA following manufacturer's protocol (R&D Systems).

For nitrotyrosine quantification, 50,000 neutrophils were lysed in RIPA buffer supplemented with a protease inhibitor cocktail (Halt protease inhibitor, Thermofosher) for 20 min at 4 °C, followed by centrifugation at 20,000g for 15 min at 4 °C. Supernatants were collected and stored at −80 °C for subsequent protein quantification by BCA and nitrotyrosine ELISA according to manufacturer's protocol (Abcam).

In vitro transmigration
Neutrophils were isolated from blood using Polymorphprep (Fisher Scientific) and transmigrated through a small airway epithelial layer (H441, ATCC) *in vitro* towards CFASN (pathological milieu) or leukotriene B_4 (LTB_4, chemoattractant control) as previously described (1). For EGFR expression measurements by flow cytometry, transmigrated neutrophils were washed twice with RPMI and phenotyped as described above. For functional assays, $2x10^6$ transmigrated neutrophils were treated with 1ng/mL EGF and/or 10µM Gefitinib for 2 hours in RPMI, washed and used for downstream assays. NOS blockade was performed with L-NAME (eNOS, 39nM), L-NMMA (nNOS, 4.1µM), or 1400W 2HCl (iNOS, 7nM), individually or combined, with or without EGF stimulation.

RNA sequencing
RNA sequencing data were obtained from a publicly available dataset on GEO Accession viewer at GSE167069 (2), where healthy control neutrophils were transmigrated into LTB_4 or CFASN for 10 hours. Analysis of transcriptomics data was performed using hisat2 with the reference genome GRCh39. Differential gene expression was determined using DESeq2 and the significance threshold was set at 0.01 for the adjusted p value (Benjamini-Hochberg correction).

Calcium signaling measurements
A black-walled, clear bottom 96-well plate (Thermo Scientific) was pre-coated with 20µg/mL of human plasma fibronectin (MilliporeSigma) for 1 hour at 37°C prior to cell seeding. During this incubation period, Calbryte™ 520 AM loading and reading solutions were freshly prepared. Loading solution was Hanks' Balanced Salt Solution without calcium or magnesium ($HBSS^{-}/^{-}$; Gibco) supplemented with 10% fetal bovine serum (FBS), 5 µM Calbryte 520 AM (AAT Bioquest, Cat. No. 20650), and 2 mM probenecid (Invitrogen). Reading solution was HBSS containing calcium and magnesium ($HBSS^{+}/^{+}$; Gibco) supplemented with 2 mM probenecid. Human neutrophils were isolated from peripheral blood and centrifuged at 500g for 5 minutes. The supernatant was discarded, and cell pellets were resuspended in the loading solution to a final concentration of $2.5x10^6$ cells/mL. Cells were incubated at 37°C for 30 minutes on an orbital shaker, protected from light. Following incubation, cells were centrifuged at 500g for 5 minutes, and the supernatant was discarded. The cell pellet was then resuspended in the reading solution. Neutrophils were subsequently seeded on to the fibronectin coated 96-well plate ($2.5x10^5$ cells/well) and treated with either vehicle control (DMSO) or 50µM BAPTA-AM (Invitrogen) for 30 minutes at 37°C. Afterwards, calcium flux was assessed using a fluorescence plate reader (excitation/emission: 490/525 nm) following stimulation with EGF at final concentrations of 0.25 or 0.5ng/mL.

Bacterial killing assays
P. aeruginosa (strain PAO1, Perkin Elmer) or *S.aureus* (strain Xen29, Perkin Elmer) were grown to reach the exponential phase. Bacteria were then incubated in RPMI and 10% heat-inactivated FBS for 30 minutes at 37°C. Transmigrated neutrophils were resuspended in RPMI, 10% heat-inactivated FBS and incubated at 37°C, 5% CO_2 for 15 minutes. Bacteria and neutrophils were co-incubated at a multiplicity of infection (MOI) of 1 for 1 hour on an end-over-end rotating wheel at 37°C. To discern between intracellular and extracellular killing, cells were spun at 800g, 10 minutes to remove the supernatant and gently lysed in 0.1% Triton-X as a first step for serial dilutions. Colony forming units (CFU) were used to determine the bactericidal capacity of neutrophils, with bacteria plus RPMI,10% FBS condition set as 100% viability.
To determine the role of EGFR in bacterial killing within the CF airway microenvironment, healthy and CF neutrophils were transmigrated into CFASN for 4 hours. Transmigrated neutrophils were co-incubated with PAO1 in CFASN in the presence or absence of Gefitinib (10µM) for 1 h at 30°C

on an end-to-end rotary shaker at a MOI of 1. Following co-incubation, cells were lysed with 0.1% Triton X-100, and lysates were serially diluted in PBS in untreated 96-well flat-bottom plates. A 5μL aliquot from each dilution was plated onto LB agar plates and incubated for 14–16 h at 37°C. Colony-forming units (CFU) were enumerated to determine the bactericidal capacity of neutrophils under each condition and were compared to PAO1-Gefitinib controls lacking neutrophils.

Statistics
Descriptive statistics (all data are shown as median and interquartile range) were assessed using Kruskal-Wallis with Dunn's multiple comparison test, Mann-Whitney's test, or by Wilcoxcon signed-rank test. All statistical tests were two-tailed. A P value less than 0.05 was considered significant. Data were analyzed using GraphPad PRISM Software Inc.

Study approval
Patient samples were obtained after informed consent on Institutional Review Board approved protocols at the University of Alabama at Birmingham (IRB-140414004) and at Emory University (IRB-00042577).

Data availability
As mentioned above, the RNA sequencing dataset is available on GEO Accession viewer at GSE167069. All other data are available upon request to the corresponding author.

Table S1. Patient demographics. AA: African American, AW: sputum, CA: Calcium signaling assay, CF: cystic fibrosis patient, FC: Flow cytometry, HD: healthy donor, HO: homozygous ΔF508, HT: heterozygous ΔF508, IM: Image cytometry, OT: other, p-ELISA: ELISA for phospho-EGFR, n-ELISA: ELISA for nitrotyrosine, TM: transmigrated neutrophils used for bacteria clearance assay, WB: whole blood.

Patient	Sex	Age	Ethnicity	CFTR genotype	Modulator therapy	Sample	Outcome
CF01	M	30	Caucasian	HO	Lumacaftor/ Ivacaftor	WB, AW	FC, IM
CF02	M	32	Caucasian	HO	None	WB, AW	FC, IM
CF03	M	20	Caucasian	HT	None	WB, AW	FC, IM
CF04	F	30	Caucasian	HO	Lumacaftor/ Ivacaftor	WB, AW	FC, IM
CF05	M	30	Caucasian	HO	None	WB, AW	FC, IM
CF06	F	28	Caucasian	OT	None	WB, AW	FC
CF07	F	26	Caucasian	OT	None	WB, AW	FC
CF08	F	52	Caucasian	HO	Trikafta	WB	FC
CF09	M	37	Caucasian	HO	Trikafta	WB	FC
CF10	F	47	Caucasian	HT	Trikafta	WB	FC
CF11	F	30	Caucasian	HO	Trikafta	WB	FC
CF12	F	47	Caucasian	HO	Trikafta	WB	FC
CF13	F	24	Caucasian	HO	Trikafta	WB	FC
CF14	M	40	AA	HO	Trikafta	WB	FC
CF15	F	47	Caucasian	OT	Trikafta	WB	FC
CF16	M	48	Caucasian	HO	Trikafta	WB, AW	FC
CF17	F	21	Caucasian	HO	Trikafta	WB, AW	FC
CF18	M	47	Caucasian	OT	Trikafta	WB, AW	FC
CF19	F	51	Caucasian	HO	Trikafta	AW	FC
CF20	M	34	Caucasian	HO	Trikafta	AW	FC
CF21	F	32	Caucasian	OT	Trikafta	AW	FC
CF22	F	31	Caucasian	HO	Trikafta	AW	FC
CF23	F	31	AA	HT	Trikafta	AW	FC
CF24	M	27	Caucasian	HO	Trikafta	AW	FC
CF25	F	33	Caucasian	HO	Trikafta	AW	FC
CF26	M	27	Caucasian	HO	Trikafta	AW	FC
CF27	F	39	Asian	OT	Trikafta	AW	FC
CF28	M	31	Caucasian	HO	Trikafta	AW	FC
CF29	F	27	Caucasian	HO	Trikafta	AW	FC
CF30	M	34	Caucasian	HO	Trikafta	AW	FC
CF31	M	47	Caucasian	HO	Trikafta	AW	FC
CF32	M	54	Caucasian	HT	Trikafta	WB	TM, CA
CF33	F	23	Caucasian	HT	Trikafta	WB	TM, CA
CF34	F	29	Caucasian	HT	Trikafta	WB	TM, CA
CF35	F	32	Caucasian	HT	None	AW	ELISA
CF36	M	28	Caucasian	HO	None	AW	ELISA
CF37	F	59	Caucasian	HT	None	AW	ELISA
CF38	F	44	Caucasian	HO	None	AW	ELISA
CF39	M	39	Caucasian	HO	None	AW	ELISA
CF40	M	64	Caucasian	OT	None	AW	ELISA

CF41	M	33	Caucasian	HO	None	AW	ELISA
CF42	M	32	Caucasian	HT	None	AW	ELISA
CF43	F	34	Caucasian	HT	Trikafta	WB	TM, n-ELISA
CF44	M	40	Caucasian	HO	Trikafta	WB	TM, n-ELISA
HD1	F	27	Caucasian	NA	None	WB	TM, FC, p-ELISA
HD2	M	24	Caucasian	NA	None	WB	TM, FC, p-ELISA
HD3	F	25	Caucasian	NA	None	WB	TM, FC, p-ELISA
HD4	M	23	Caucasian	NA	None	WB	TM
HD5	M	40	Caucasian	NA	None	WB	TM
HD6	F	25	Caucasian	NA	None	WB	TM
HD7	M	22	Caucasian	NA	None	WB	TM, n-ELISA

Figures

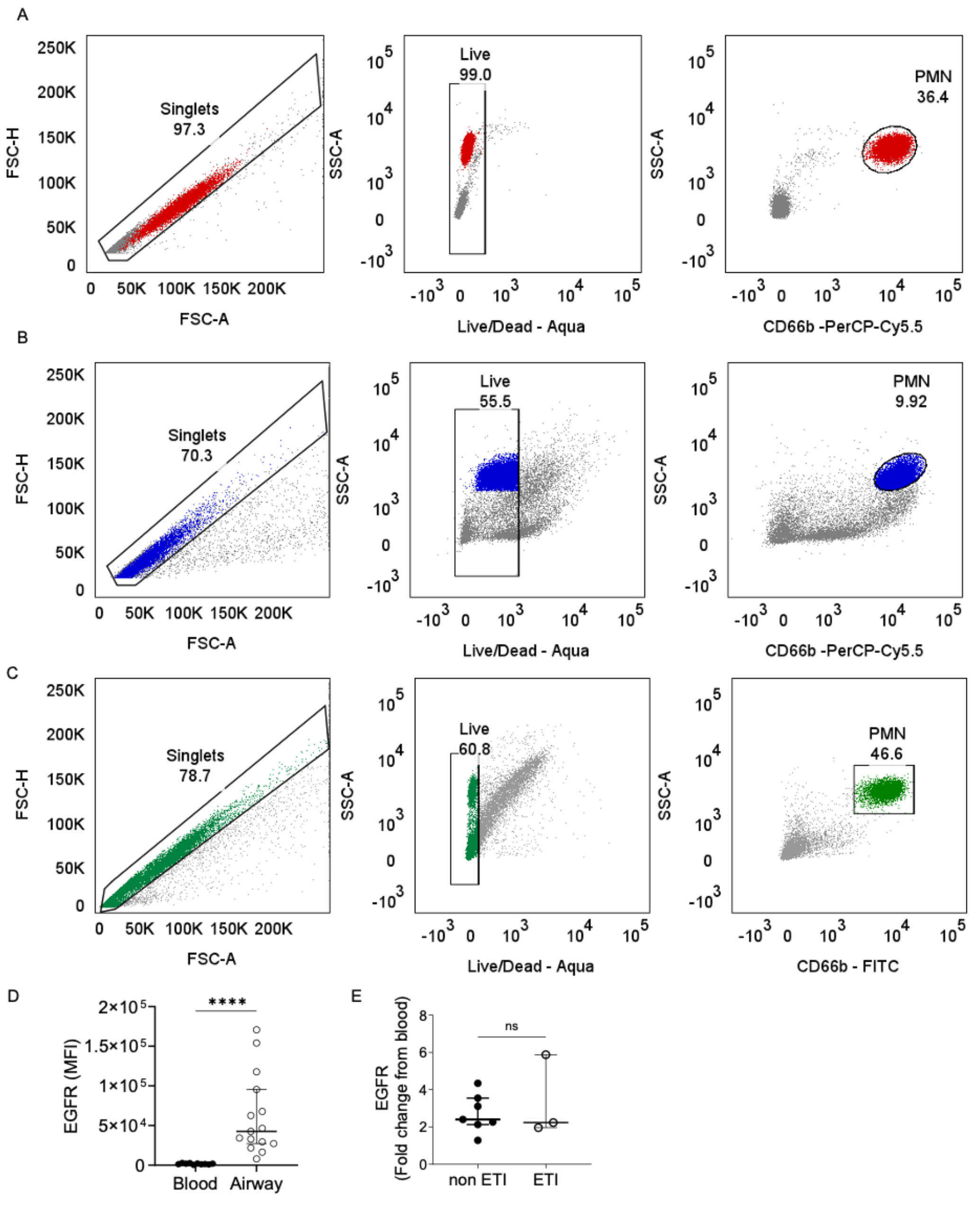

Figure S1. Gating strategy for flow cytometry analysis.
Representative flow cytometry analysis of blood **(A)** and airway **(B)** neutrophils from a patient with CF, and a healthy control neutrophil sample following *in vitro* transmigration **(C)**. **(D)** Median fluorescence intensity (MFI) of surface EGFR in blood and airway CF neutrophils patients treated with Trikafta. **(E)** Comparison of EGFR expression in airway neutrophils from patients not on ETI and from CF patients on ETI. Statistical analysis was performed with Mann-Whitney test, **** $p < 0.0001$, ns - not significant.

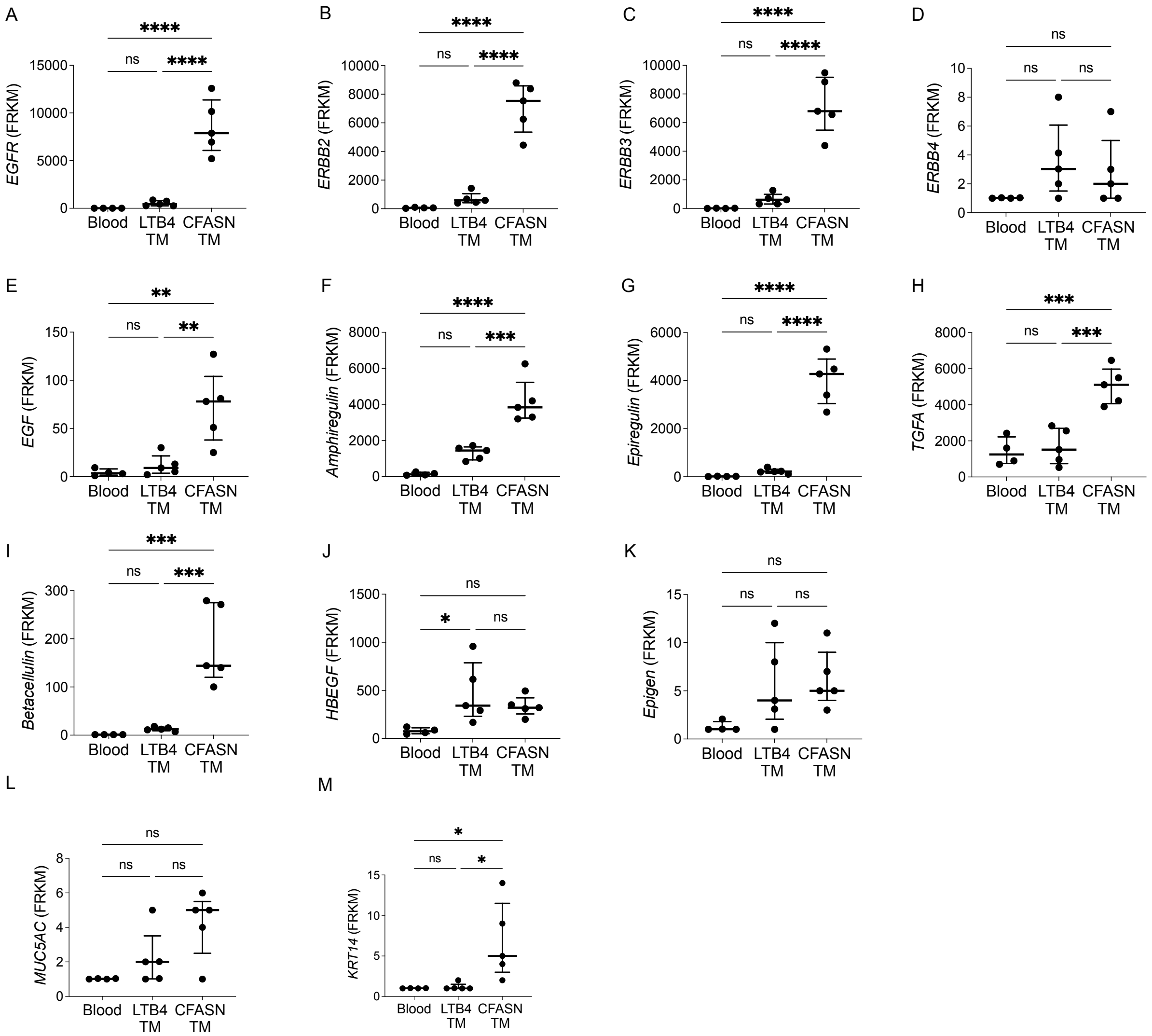

Figure S2. CF airway neutrophils express RNA for EGFR and its ligands. RNA sequencing analysis of blood neutrophils, as well as control transmigrated (LTB4 TM), and CFASN transmigrated (CFASN TM) healthy control neutrophils, shows increased expression of EGFR and other ERBB receptors **(A-D)** and EGFR ligands **(E-K)**. **(L-M)** Expression of epithelial genes was not different between groups. Statistical analysis was performed using one-way ANOVA with Tukey's correction for multiple comparison test. Ns = not significant, *p<0.05, **p<0.01, *** p<0.001, ****p<0.0001.

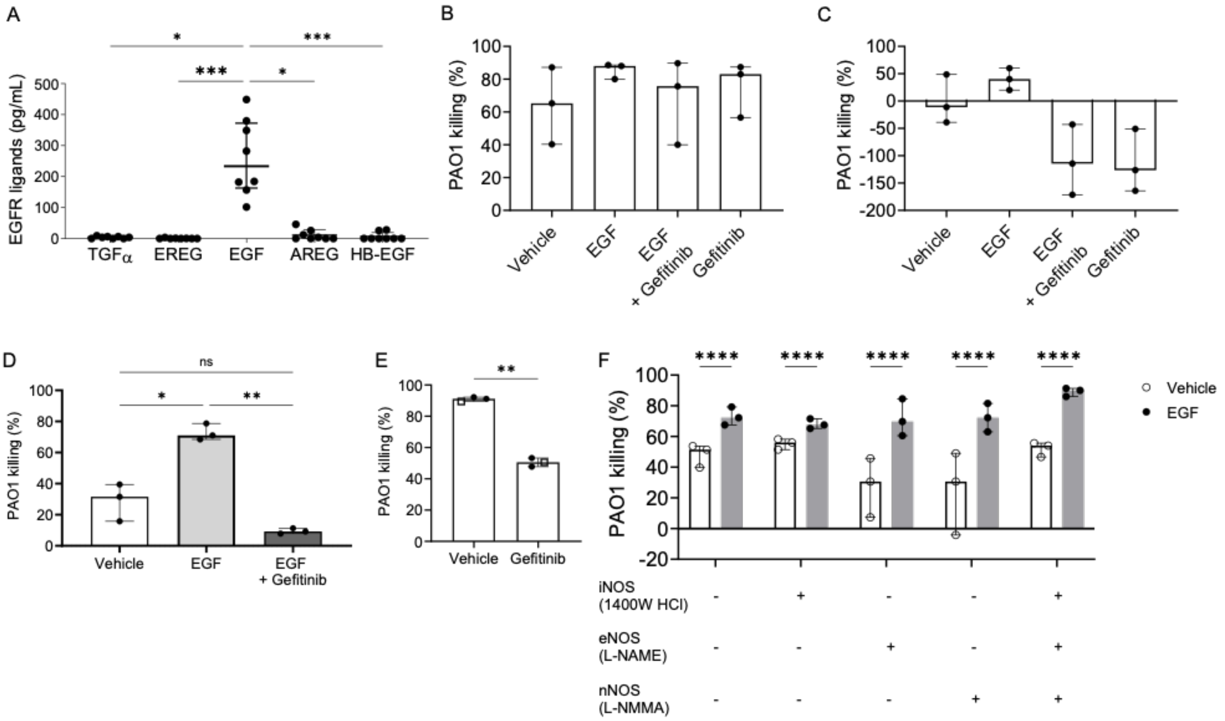

Figure S3. EGF stimulation of CF lung-conditioned neutrophils increases bactericidal activity in vitro. (A) EGFR ligands in sputum supernatant from CF patients (N=8) were quantified by ELISA (EREG = epiregulin, AREG = amphiregulin). **(B)** Extracellular bacterial clearance after 2 hour stimulation with EGF of healthy control neutrophils transmigrated into CFASN. **(C)** Intracellular bacterial clearance after 2 hour stimulation with EGF of healthy control neutrophils transmigrated into CFASN. **(D)** PAO1 clearance after EGF stimulation of CF blood neutrophils transmigrated into CFASN. **(E)** Neutrophils were migrated into CFASN and PAO1 clearance was performed within CFASN with EGFR blockade by Gefitinib. (Dots = CF neutrophils transmigrated into CFASN, open square = healthy control neutrophils transmigrated into CFASN). **(F)** Bacterial clearance after 2 hour stimulation of healthy control neutrophil transmigrated into CFASN with EGF and NOS blockade. Statistical analysis was performed using Kruskal-Wallis with Dunn's multiple comparison test (panels A-D), Wilcoxon signed-rank test (panel E), or two-way ANOVA (panel F). *$p<0.05$, **$p<0.01$, ***$p<0.001$, ****$p<0.0001$.
